# The Athletic ECG: A Line of Defense Against Misinterpretation

**DOI:** 10.1016/j.jaccas.2022.08.024

**Published:** 2022-11-16

**Authors:** Rahul Ghelani, Ji-Jian Chow, Amanda Varnava

**Affiliations:** a Chelsea and Westminster National Health Service Foundation Trust, London, United Kingdom; b Cardiology Department, Imperial College Healthcare Trust, London, United Kingdom

**Keywords:** cardiac risk, echocardiography, electrocardiogram, exercise, imaging, myocarditis, sports cardiology, CMR, cardiac magnetic resonance, ECG, electrocardiogram, EMB, endomyocardial biopsy

## Abstract

A 17-year-old competitive athlete was found to have a minor electrocardiogram abnormality on routine screening. Cardiac magnetic resonance revealed evidence of marked myocarditis, allowing a subsequent safe abstinence from exercise. The case highlights the importance of careful electrocardiogram interpretation, especially in athletes, where physiologic adaptive changes can pose a diagnostic challenge. (**Level of Difficulty: Intermediate.**)

## Central Illustration

**Figure:**
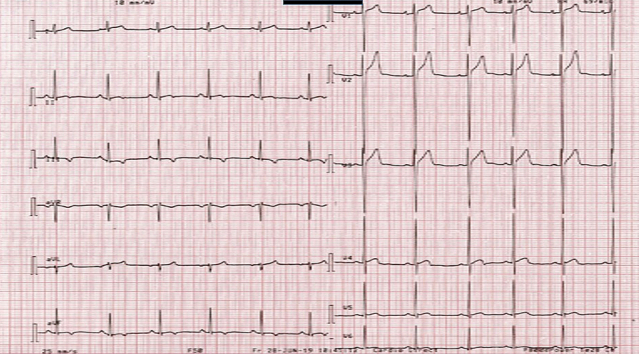

## History of Presentation

**Figure 1:**
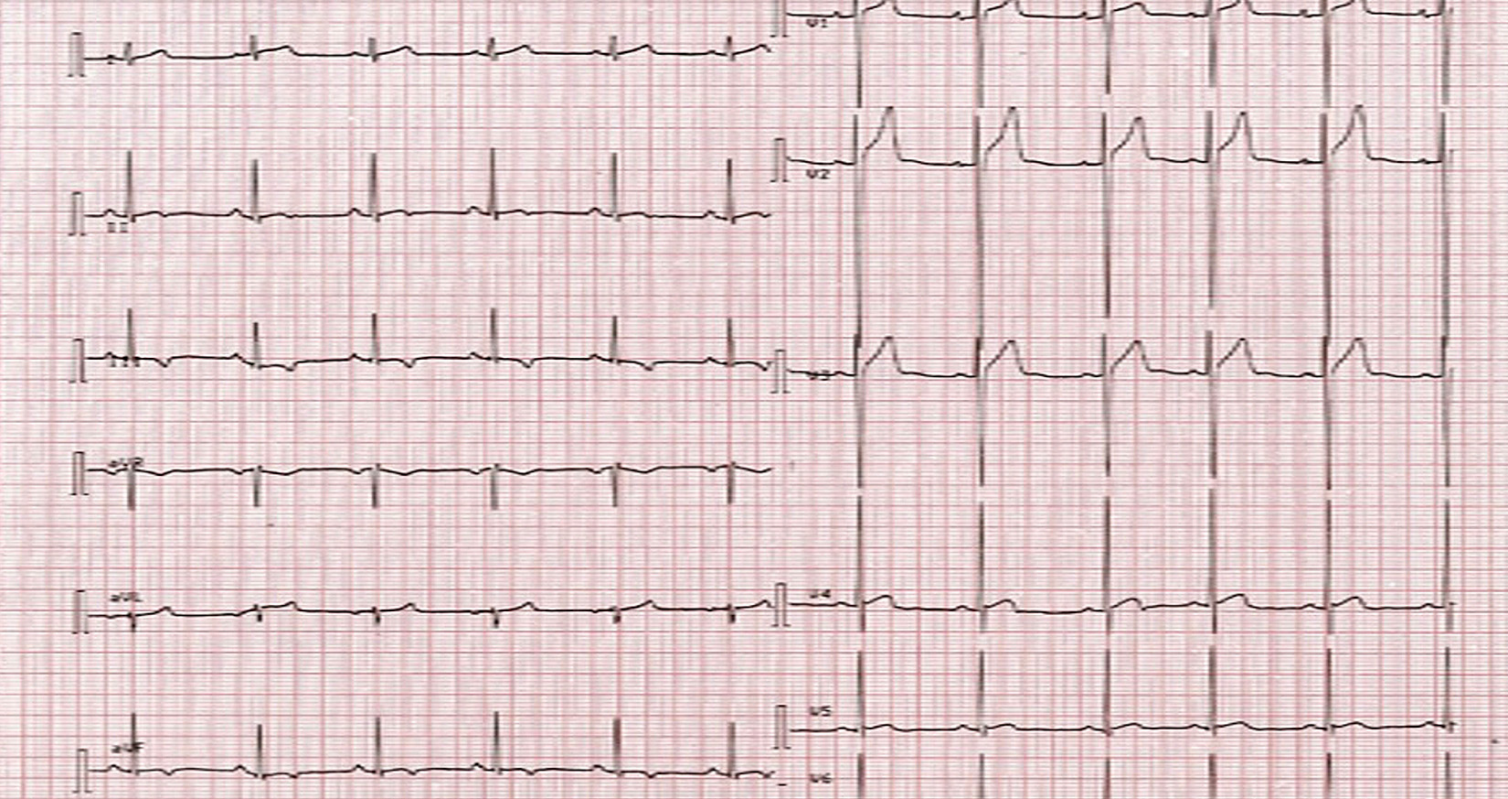
Initial Electrocardiogram on Presentation T-wave inversions seen in aVR, lead III (potentially normal variants but new compared to baseline electrocardiograms), and aVF.

We describe the case of an asymptomatic 17-year-old male African Caribbean competitive soccer player (training 4-6 h/d) with an abnormal resting electrocardiogram (ECG) seen on pre-competition screening (Figure 1). T-wave inversions were seen in aVR, aVF, and lead III (new compared to baseline ECGs) and an early repolarization was seen in leads V_1_ through V_3_. Conduction intervals were normal. He had excellent exercise tolerance and reported no chest pain, palpitations, presyncope, syncope, or a recent history of infection symptoms. Physical examination findings were unremarkable.

**Learning Objectives**

• To bridge the challenge of distinguishing ECG findings suggestive of a potentially serious cardiovascular disorder from benign physiologic adaptations occurring as a result of regular intense exercise.
• To understand the complexity involved in advising an athlete to return to competitive sports in the context of cardiovascular disease.

## Past Medical History

There was no personal or family history of premature cardiac disease or sudden cardiac death. He took no prescribed medications or recreational drugs.

## Differential Diagnosis

The “hot phase” of an arrhythmogenic cardiomyopathy, myocarditis, and cardiac sarcoidosis were considered.

## Investigations

**Figure 2:**
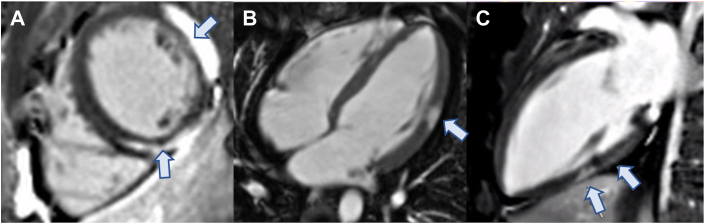
Initial Cardiac Magnetic Resonance Images on Presentation **(A to C)** Cardiac magnetic resonance images suggestive of myocarditis, with subepicardial late gadolinium enhancement in the midlateral wall and midwall late gadolinium enhancement in the midinferior wall **(arrows)**.

Transthoracic echocardiography was normal. In contrast, cardiac magnetic resonance (CMR) demonstrated high T1 and T2 parametric mapping values with corresponding late gadolinium enhancement (LGE) (Figure 2) in the midlateral and midinferoseptal walls of the left ventricle, suggestive of active myocardial inflammation. There was no fatty infiltration suggestive of arrhythmogenic right ventricular cardiomyopathy. Biventricular function was preserved. These findings were most consistent with active myocarditis.

High-sensitivity troponin I level was 485 ng/L (normal: 0-14 ng/L). Serum angiotensin-converting enzyme level was 63 nmol/mL/min (normal: 8-52 nmol/mL/min), normalizing to 50 nmol/mL/min on repeat testing. Parvovirus serology results were positive. Results from the remainder of a cardiotropic viral serology screen, *Borrelia* antibody level, tuberculosis enzyme-linked immunospot, serum immunoglobulin, antinuclear antibodies, antineutrophil cytoplasmic antibodies, extractable nuclear antigens, erythrocyte sedimentation rate, C-reactive protein, and genetic testing for cardiomyopathy were normal (presentation was before 2020 and COVID-19 was not considered).

Fluorodeoxyglucose cardiac positron emission tomography revealed inflammation within lateral and inferolateral aspects of the heart and significant uptake within 2 axillary lymph nodes. Axillary lymph node biopsy samples revealed reactive changes only. Endomyocardial biopsy (EMB) was considered but not performed because of concerns about complications.

**Figure 3:**
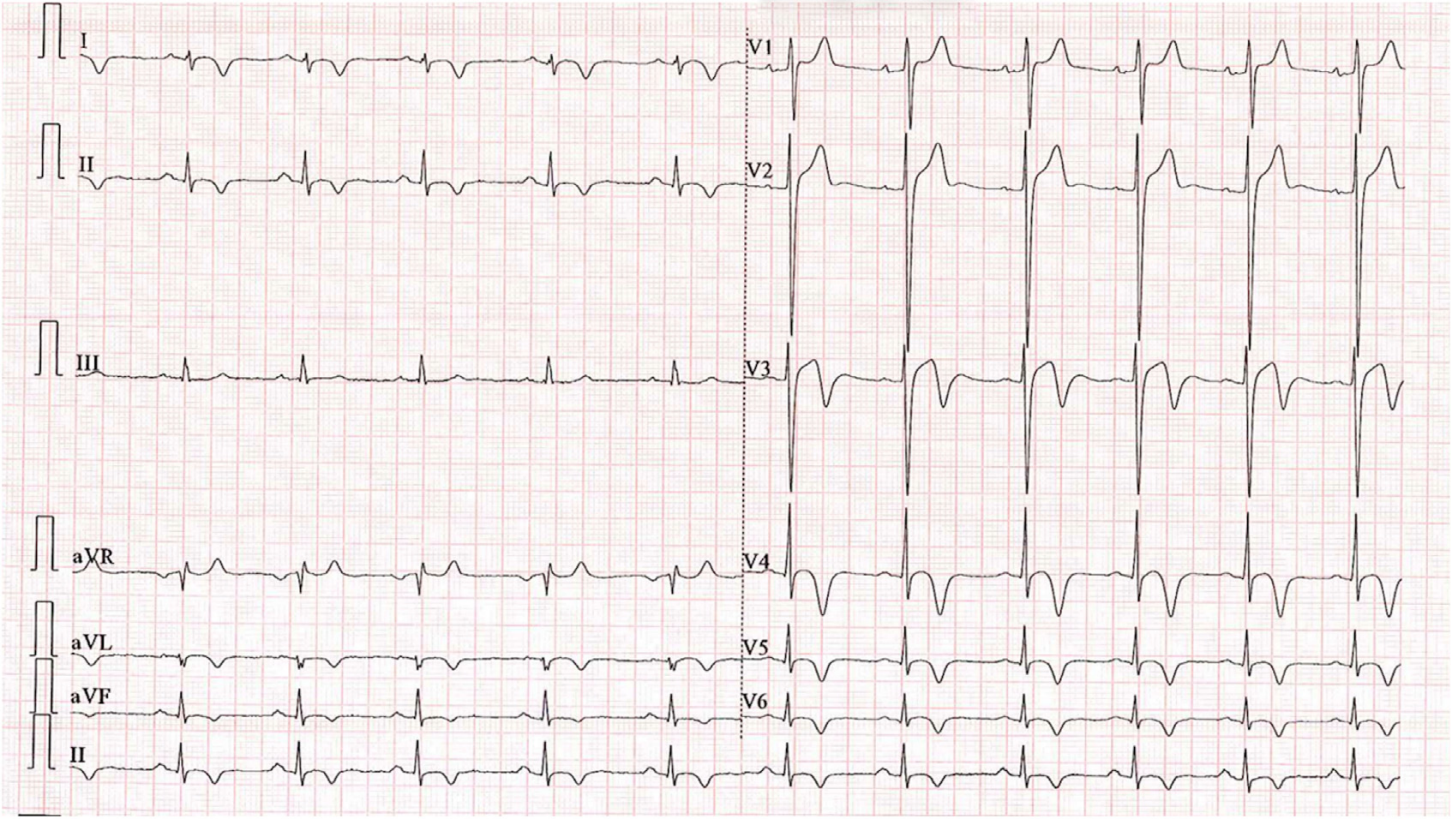
New Anterior T-Wave Inversion in Anterior-Lateral Leads, Lead II, and aVF on Electrocardiogram

Despite the patient remaining asymptomatic, serum troponin level rose to 10,185 ng/L, suggesting worsening myocardial injury. The ECG also developed new deep T-wave inversions in the anterior-lateral leads (Figure 3). The athlete was admitted for cardiac monitoring; over a week, serum troponin normalized. However, repeat CMR revealed significant new areas of inflammation with high T1 and T2 values. Late gadolinium enhancement was noted apically, while biventricular function remained normal.

## Management

It is well established that avoiding strenuous exercise is important because it can provoke life-threatening arrythmias in the context of inflammatory heart disease. European Society of Cardiology guidelines advise that return to competitive sports can be considered after 3 to 6 months in asymptomatic individuals; our patient was advised to avoid intense exertion for 6 months.^1^, ^2^, ^3^

Prednisolone 30 mg once daily was started empirically, with a key differential including cardiac sarcoid. A week into therapy, serial troponin, transthoracic echocardiography and 24-hour continuous ECG monitoring findings showed no abnormalities. At week 6, CMR showed significant improvement in the amount of myocardial inflammation (with near-normal T2 parametric mapping values). Extensive epicardial LGE however remained in one-third of the nonseptal left ventricle, potentially representing scarring.

**Figure 4:**
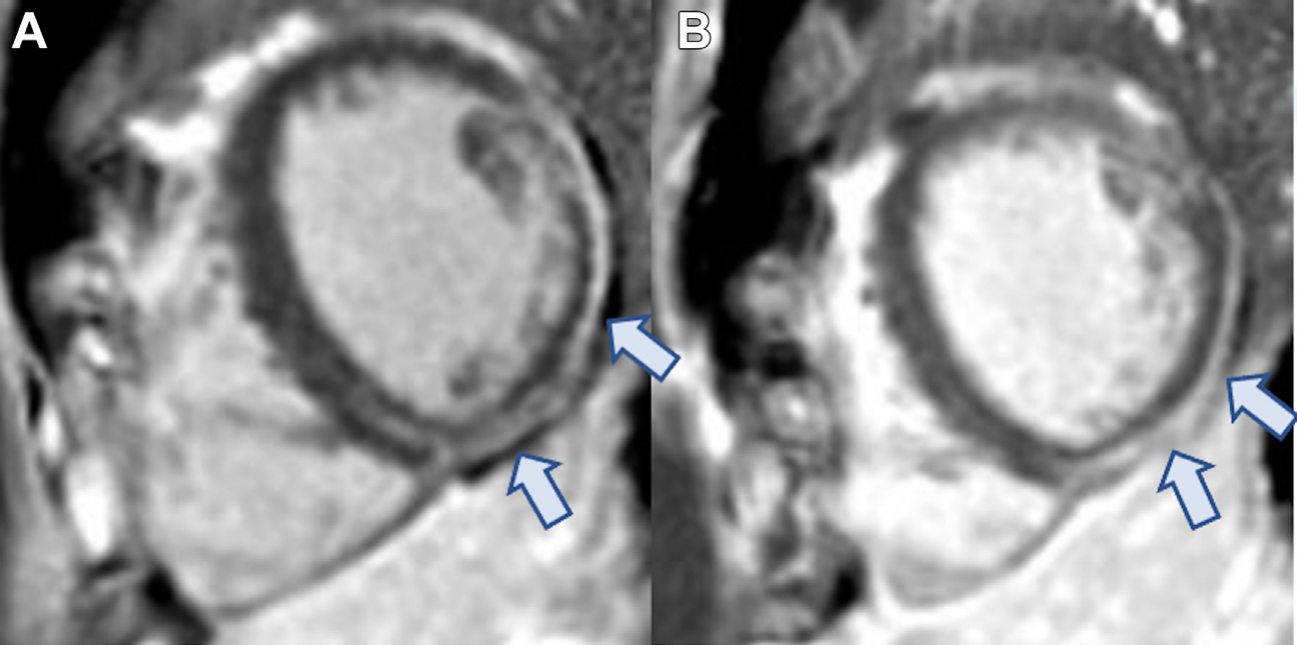
Interval Cardiac Magnetic Resonance Revealing Significant Improvement in Previously Seen Abnormalities **Arrows** indicate regions with late gadolinium enhancement.

After 12 weeks of steroids, CMR demonstrated further improvement in active inflammation with one small remaining patch of raised T2 values but persistence of epicardial LGE (Figure 4). Findings from 24-hour continuous ECG remained unremarkable. Prednisolone was subsequently weaned by 5 mg every 14 days.

**Figure 5:**
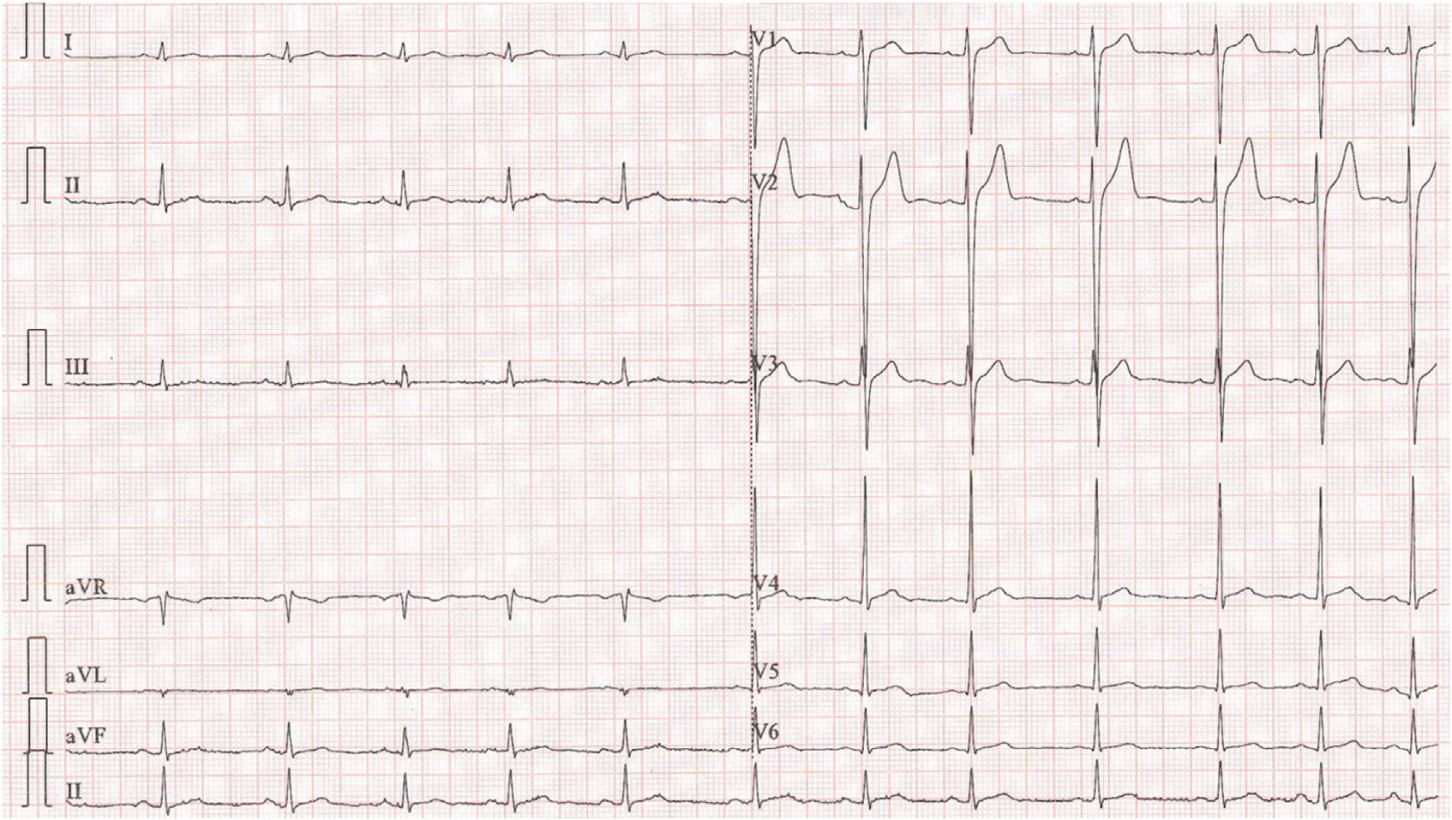
Normalization of Electrocardiogram to Baseline

Following completion of steroid treatment, serial 12-lead ECG findings were normal (Figure 5). Exercise ECG, continuous ECG patch monitoring during training and serial troponin measurements were unremarkable. Further CMR showed no further changes.

## Discussion

Sudden cardiac death is the leading cause of mortality in athletes during sports and many of the underlying causes can be identified by ECG abnormalities.^4^ However, distinguishing cardiovascular pathology from benign physiologic adaptation associated with high-volume exercise can be challenging. Guidance can be obtained from sources including the European Society of Cardiology and the Seattle criteria. Our patient’s T-wave inversions were subtle but fell outside the international criteria for benign ECG variants in athletes, highlighting the need for careful interpretation.

Myocarditis treatment focuses on managing complications such as heart failure and arrhythmia, along with etiology-targeted therapy.^1^ A clear cause was not identified in this case, which limits therapeutic options. The significance of a positive result on serum Parvovirus titer is limited by its high prevalence in the general healthy population; viral genome found on EMB would be more specific.^1^

Our patient was a competitive athlete, desiring early return to competition. Clinicians should be mindful of the effect of exercise restriction on career and well-being; fortunately, he completed the restriction period without ill effect. Immunomodulatory therapy, most commonly steroids, is an option typically in EMB-proven autoimmune, infection-negative myocarditis.^1^ Although EMB may not always be preferred, proactive management may still be required, especially when cardiac damage appears progressive—a concern in this case. Our patient recovered well, but trials using steroids for etiology-unknown myocarditis have shown a mix of favorable and neutral results. Current guidelines suggest a case-by-case consideration.^1^ Initial positron emission tomography/computed tomography and CMR suggested cardiac sarcoid as a differential and before the lymph node biopsy result, a shared decision between the patient and multidisciplinary team was taken to proceed with empiric therapy. This highlights a common ethical scenario where the diagnosis can be unclear but expert opinion and patient wishes lead to the offering of treatment, with a potential acknowledged risk.^5^

A risk assessment of future professional sports was carried out following the completion of treatment. Reassuringly, the patient was asymptomatic with excellent functional capacity and had normal biventricular function and exercise ECG testing results. The use of high-sensitivity troponin for assessing inflammatory myocardial disease can be challenging in athletes because of confounding physiologic rises after exercise. Accurate risk assessment therefore required the consideration of multiple pieces of evidence, especially imaging. All investigations suggested quiescent myocarditis, but the significance of the LGE seen on CMR and its implications for returning to sports remained unknown. Most of the research guiding the decision making in this scenario draws on expert opinion in the absence of large trial data.^2^ The latest guidance post-dates our case and suggests that those with extensive myocardial scar (>20% LGE) should abstain from moderate- to high-intensity sports.

## Follow-Up

A national soccer cardiology expert panel was consulted. They concluded that a risk would always exist but that strategies to minimize this could allow an acceptably safe return to full competitive sports after 6 months from the initial presentation. This included ensuring the availability of resuscitation equipment and trained providers, along with mobile cardiac monitoring.

The athlete underwent cardiac monitoring during routine training, which detected no arrhythmia and he remained asymptomatic. Monitoring was repeated every 3 months. Repeat CMR 6 months after the completion of corticosteroid treatment showed that the epicardial ring of LGE had faded. After extensive risk counseling regarding re-engaging in competitive sports, a shared decision was made between the athlete and multidisciplinary team to allow a return to playing sports. He has continued to compete safely to this day with close surveillance for arrhythmia or evolution of a progressive arrhythmogenic cardiomyopathy.

## Conclusions

The introduction of the 2017 athletic ECG guideline has simplified assessment for the general physician. We, however, highlight how even subtle ECG changes can be associated with significant myocardial damage, demonstrating the care needed in interpretation. The case depicts the investigative process to diagnose myocarditis along with the challenges of management when the etiology is unclear. Safely advising a return to playing sports following cardiac insult is also complex, necessitating the advice of a multidisciplinary team.

## Funding Support and Author Disclosures

The authors have reported that they have no relationships relevant to the contents of this paper to disclose.
